# Comparison of HPLC and NMR for quantification of the main volatile fatty acids in rumen digesta

**DOI:** 10.1038/s41598-021-03553-9

**Published:** 2021-12-21

**Authors:** Mengyuan Wang, Haiying Wang, Huiru Zheng, Dusan Uhrin, Richard J. Dewhurst, Rainer Roehe

**Affiliations:** 1 School of Computing, Ulster University, Belfast, UK; 2 EaStCHEM School of Chemistry, University of Edinburgh, Edinburgh, UK; 3 Scotland’s Rural College, Edinburgh, UK

**Keywords:** Metabolomics, Metabolomics

## Abstract

Accurate quantification of volatile fatty acid (VFA) concentrations in rumen fluid are essential for research on rumen metabolism. The study comprehensively investigated the pros and cons of High-performance liquid chromatography (HPLC) and ^1^H Nuclear magnetic resonance (^1^H-NMR) analysis methods for rumen VFAs quantification. We also investigated the performance of several commonly used data pre-treatments for the two sets of data using correlation analysis, principal component analysis (PCA) and partial least squares discriminant analysis (PLS-DA). The molar proportion and reliability analysis demonstrated that the two approaches produce highly consistent VFA concentrations. In the pre-processing of NMR spectra, line broadening and shim correction may reduce estimated concentrations of metabolites. We observed differences in results using multiplet of different protons from one compound and identified “handle signals” that provided the most consistent concentrations. Different data pre-treatment strategies tested with both HPLC and NMR significantly affected the results of downstream data analysis. “Normalized by sum” pre-treatment can eliminate a large number of positive correlations between NMR-based VFA. A “Combine” strategy should be the first choice when calculating the correlation between metabolites or between samples. The PCA and PLS-DA suggest that except for “Normalize by sum”, pre-treatments should be used with caution.

## Introduction

Volatile fatty acids (VFAs) produced by rumen fermentation are the most important energy source for ruminant animals^1^. Rumen VFAs play an important role in the carbohydrate nutrition of ruminants and have very close relationships with the composition and dynamics of the rumen microbial community. Furthermore, the proportions of individual VFA reflect different metabolic pathways. Propionate is the main source of gluconeogenesis, while acetate and butyrate are substrates for long-chain fatty acid synthesis^2^. The acetate to propionate ratio is strongly correlated with the highly potent greenhouse gas methane emissions from ruminants^3,4^. Ruminal branched-chain VFA (isovalerate and isobutyrate), valerate and ammonia are products of ruminal protein degradation^5^. To understand the metabolism of rumen microbes and provide relevant numerical support for animal nutrition studies, accurate quantifications of rumen VFA are needed.

The standard methods for quantification of VFA include chromatography (gas phase or liquid phase), electromigration (capillary isotachophoresis), and spectrophotometric methods^6^. The traditional distillation process with subsequent titration has poor selectivity and productivity and is no longer widely used^7^. There have been reports of colourimetric methods applied to the analysis of organic acids, but which can only measure single acids^8^. Therefore, the chromatographic approaches of identification are more common.

Rumen VFA were first measured by high-performance liquid chromatography (HPLC) in 1988^9^ and HPLC is a simple, and reliable method to quantify rumen VFA. Nowadays, HPLC has been widely used in medicine, biochemistry, environmental protection, agriculture, and other scientific fields based on its many advantages. HPLC can analyse more than 70% of organic compounds^10^. The average time to analyse a sample is 15–30 min, but some samples can be analysed in less than 5 min, whereas others take up to an hour^11^. HPLC also has the benefit of allowing the chromatographic column to be reused and causing no damage to the sample.

HPLC also has its shortcomings. It is expensive, requires various packing columns, has a small capacity, is not well suited to analyse biological macromolecules and inorganic ions, and the eluent of mobile phase consumes a lot of most toxic chemicals^12^. Although HPLC is commonly used in the analysis of biological samples, the majority of applications are for targeted component analysis rather than overall metabolite fingerprinting^13,14^.

As early as 1999, Attaelmannan et al. used proton nuclear magnetic resonance spectroscopy (NMR) to determine the main VFAs in rumen fluid^15^. Concentrations of acetate, propionate and butyrate in the rumen measured by NMR were not significantly different from the values determined by gas chromatography. NMR has several advantages for metabolite profiling: it is non-destructive, does not require chromatographic separation and minimal sample preparation is sufficient^16^. In addition, as a quantitative analytical technique that NMR can provide structural information about unknown compounds. The intensity of NMR signals is proportional to the concentration of compounds. This feature maximizes the information content of NMR spectra, even though excessive overlap of NMR signals can compromise this process. Matrix constituents and properties (i.e., salt, pH, additives, ionic strength) have a large effect on NMR analysis, but their variation can be controlled i.e., using buffers^17^. The fundamental disadvantage of NMR is its limited sensitivity, which typically limits to the μM range. The presence of significant peak overlap in the NMR spectra of complicated mixtures makes the measurement of low concentration metabolites difficult. The accuracy of quantification of overlapping compounds can be greatly improved by commercial software (such as the Chenomx NMR Suite 8.6)^18^. One drawback that cannot be ignored is the relative high acquisition and maintenance costs of NMR instrumentation, the need for specialised staff and expensive commercial software for quantitative analysis.

Pre-processing and pre-treatment are critical and necessary procedures before downstream data analysis, such as statistical models, for each quantitative metabolite approach. Because the pre-processing/pre-treatment determine whether findings from downstream data analysis accurately represent biological fact. Variability in metabolomic samples is primarily due to two factors: Technical variability is introduced during the pre-analytical and data processing steps, while biological variability is introduced owing to variability in the samples^19^. Biological variability varies according to the biological samples being studied. Due to variations in heredity, body weight, and nutrition, biological variability in biofluids such as urine or serum can be significantly larger^20^. For metabolite quantification, the allowable technical variability should typically not exceed 20%^21^. If the variations in sample concentrations between comparison samples are higher than the analytical technical variance, sample normalisation is required in a metabolomics study. A proper pre-treatment method, according to related studies^22^, can improve the observed fit.

This study comprehensively investigates the pros and cons of HPLC and NMR analysis methods for work procedures and empirical results on the determination of ruminal VFA. Our work aimed primarily to verify the repeatability, precision, and limits of methods to provide insight into the best approach for animal nutrition research.

## Materials and methods

**Figure 1:**
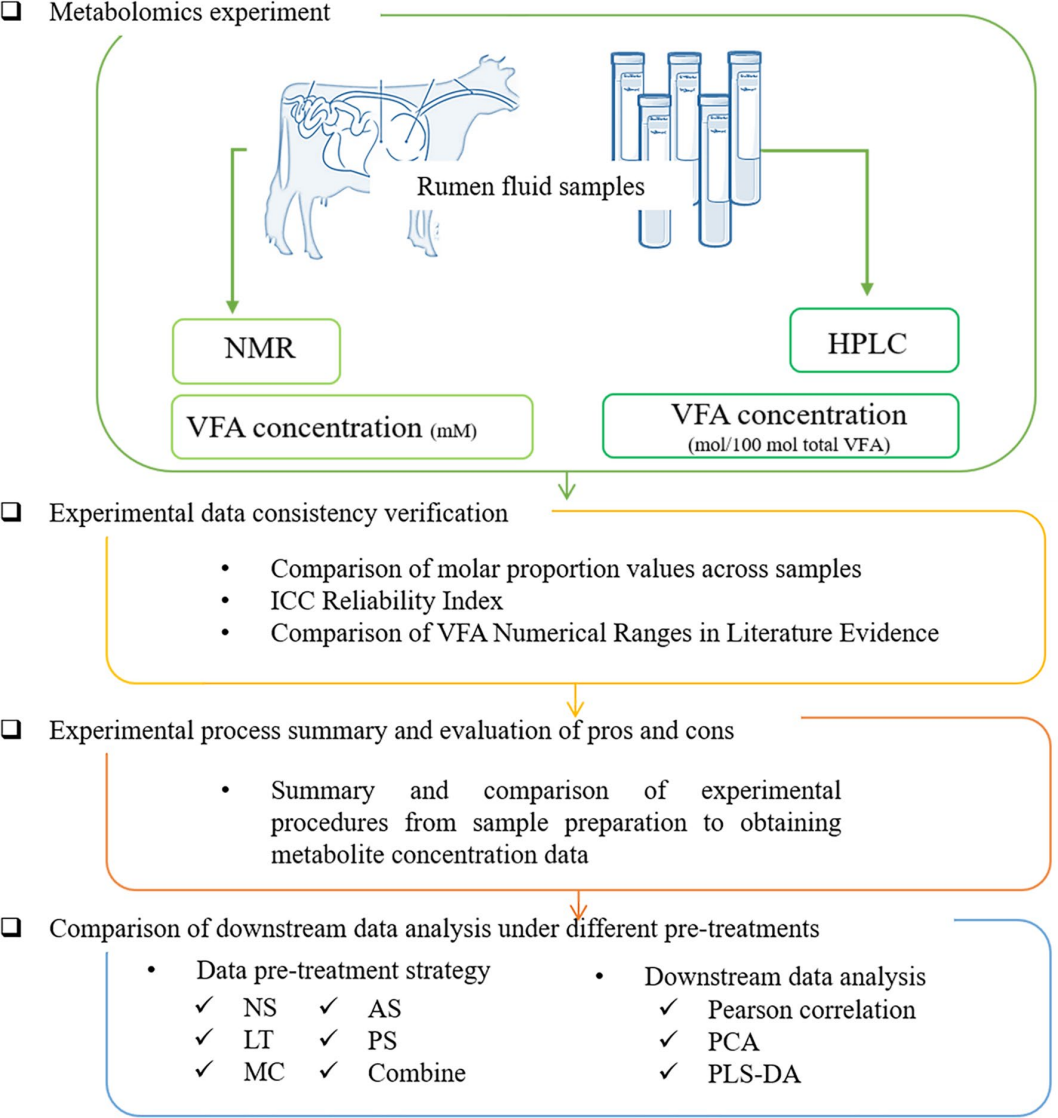
Research pipeline. The artwork used elements from Servicer Medical Art: https://smart.servier.com/.

This research follows the pipeline as shown in Fig. 1 for analysis and presentation.

### Animals, experimental design and diets

The Beef and Sheep Research Centre at Scotland's Rural College collected the data from research using beef cattle (SRUC, Edinburgh, UK). The rumen fluid samples came from a total of 33 animals, all of which were balanced in terms of breed type and diet. Samples from the experiment were designed with two diet types (forage and concentrates proportion respectively are CONC:500–500 and FOR:80–920), four breeds, and three additives by Roehe et al.^23^. The concentrations of VFAs were obtained separately by analyzing the same batch of rumen fluid samples through two experimental techniques: HPLC and NMR (Fig. 1). The beef cattle in this experiment set up duplicate groups and control groups of breed, diet and addition. In the case of balancing the ex perimental treatments of each group, 33 samples were finally used in this study^23^.

### Rumen volatile fatty acid (VFA) analysis

#### HPLC analysis

The HPLC analysis was completed by Rooke et al. in 2014, and detailed experimental procedures and methods were introduced in related publication^4,24^. Volatile fatty acids were determined by HPLC. Silage extracts were centrifuged for 5 min at 11,000*g* and 0.02 ml aliquots injected in duplicate onto a Bio-Rad HPX 87H ion-exchange column (300 × 7·8 mm) (Bio-Rad Laboratories Ltd, Watford, UK) protected by a 30 mm guard column containing a cation H^+^ ion-exchange resin. The eluting solvent was 0.005 mol l^−1^ H_2_SO_4_ and the chromatograph was operated at 50 °C at a flow rate of 0.8 ml min^−1^. Peak detection was by reference to an external standard^24^. To achieve high precision and accuracy of the VFA measurement, VFA standards were run in our study every 10th sample. It is generally known that HPLC is characterized by high precision (coefficient of variance < 2.5%) and high coefficient of determination (R^2^ > 0.997)^25^.

#### NMR analysis

The details of the performed ^1^H-NMR experiment can be found in Bica et al.^26^. Chenomx NMR Suite tool (v8.6; Chenomx, Canada; https://www.chenomx.com/) was used to identify and quantify rumen metabolites based on ^1^H-NMR spectra, as reported by Ametaj et al.^27^ and Jung et al.^28^. The method employs an internal standard such as DSS with known concentration. This reference compound is simply introduced to the sample before the experiment. The volumes of the peaks that correspond to metabolites with unknown quantities are calculated. They're subsequently compared to DSS to determine the metabolites' absolute concentrations. In fact, by comparing with the Chenomx database, in the NMR experiment, we identified and quantified 118 metabolites, including VFAs. The NMR spectrum of each VFA is shown in Fig. S1. For NMR, Dumas et al.^29^ reported high multivariate analytical reproducibility of > 98%.

### Data pre-treatment and statistical analysis

#### Reliability analysis

To evaluate whether the two methods, i.e., HPLC and NMR are consistent in the determination of the same compound, ICC-based reliability analysis was performed. First, auto-scaling is used to range-scale the two sets of HPLC and NMR data for comparison. Following the guidelines presented in^30^, to consider the systematic error of the rater, ICC two-way random/mixed, absolute agreement, multiple raters/measurements (2, A, K) model was selected for the investigation.

#### Pre-treatment measure

Based on the summarized pre-treatment methods for metabolomics data, we investigated the performance of these methods, including normalization by sum (NS), log transformation (LT), mean centring (MC), auto-scaling (AS), Pareto-scaling (PS), and the combination of normalized, Pareto-scaling and log-transformation (Combine). The details of the above pre-treatment have been summarized by Robert et al.^22^. MetaboAnalystR 3.0 were used to complete the data pre-treatment.

#### Statistical analysis

Principal component analysis (PCA), partial least-squares discriminant analysis (PLS-DA), and correlation analysis were completed on the MetaboAnalystR 3.0 platform. PLS-DA performs tenfold cross-validation on the top five principal components. The PLS-DA model was evaluated using the model-R^2^, cross-validated Q^2^, and prediction accuracy. Q^2^ as the selected criterion has the advantage that less prone to overfitting^31^. Regarding the PLS-DA model parameters, the closer the R^2^ value is to 1, the better the interpretation of the PLS-DA model; the closer the Q^2^ value is to 1, the better the prediction of the PLS-DA model^32^. Generally, R^2^ and Q^2^ are good when greater than 0.5, and more than 0.4 is acceptable^32^.

### Ethics approval and consent to participate

Each of the individual experiments in this study was approved by Scotland’s Rural College (SRUC) Animal Experiments Committee, which operates as the Local Ethical Review Group required under the UK Animals (Scientific Procedures) Act 1986. The studies were conducted at the SRUC Beef and Sheep Research Centre in Edinburgh, and all work was undertaken in accordance with ARRIVE guidelines, as well as the requirements of the UK Animals (Scientific Procedures) Act 1986.

## Results and discussion

To reveal the real metabolomic changes caused by specific biological events, it is critical to use a suitable analytical pipeline that can accurately and precisely detect the true concentration differences of individual metabolites. Metabolomic analysis entails several steps including pre-analytical work (i.e., biofluid sample collection and storage), experimental work (i.e., sample analysis) and data analysis (i.e., pre-process, quantification, and pre-treatment)^30^. Concentrations of rumen VFA measured using HPLC and NMR were compared for these samples, and the reliability of results was assessed using the intraclass correlation coefficient (ICC). Then we summarized the analytical workflows of HPLC (Fig. 3) and NMR (Fig. 4). Based on the results of this research in the pre-processing and quantification of NMR spectroscopy, some practical suggestions are given. PCA, PLS-DA and correlation analysis were used to compare the concentrations collected by the two methods after different pre-treatment measures. Relevant suggestions were provided consequently.

### Comparison of the quantitative rumen VFA data determined by HPLC and NMR

Table 1 summarizes the VFA concentration range from literature using both methods as a basis for comparison with current values. The concentration of volatile fatty acids in the rumen will vary a lot depending on the diets and individuals. For HPLC and NMR, there are potential issues about which units the VFA are reported as—concentrations or molar proportion (Fig. S2). Molar proportions of VFA in the rumen are commonly reported because they are more reliable to represent either VFA production, absorption, or both^34^. The balance of generation and removal or interconversion from the pool determine the concentration of each VFA in the rumen. Furthermore, concentrations might fluctuate depending on the time and site of sampling. VFA can be diluted in the rumen by water and saliva without changing their relative proportions^34^. It has also to be considered that in the experiment, rumen fluid was always diluted into differing liquid amounts; VFA mol or VFA molar percentages are unaffected by digesta liquid amount. Hall et al. have reported that it is more biologically meaningful when ruminal data were evaluated as moles of VFA (VFA/mol) than concentrations (mM)^35^.

**Table 1:** Comparison of experimental concentration of volatile fatty acids with literature values.

Methods (unit)	VFA	Literature value		Experimental data (mean ± SE)
		Minimum	Maximum	
Absolute concentration based on NMR (mM)	Acetate	29.68	81	35.97 ± 16.83
	Propionate	11.55	22.04	11.03 ± 5.45
	Butyrate	6.47	35.12	6.60 ± 4.18
	Isobutyrate	0.49	1.66	0.71 ± 0.35
	Isovalerate	0.68	1.3	0.83 ± 0.39
	Valerate	1.3	5	0.86 ± 0.39
Molar proportions of concentrations based on HPLC (mol/100 mol total VFA)	Acetate	49.66	69.94	63.26 ± 5.17
	Propionate	14.71	40.59	20.88 ± 6.36
	Butyrate	8.77	13.32	11.5 ± 3.02
	Isobutyrate	0.8	1.09	1.24 ± 0.41
	Isovalerate	0.65	0.89	1.57 ± 0.36
	Valerate	0.78	1.05	1.57 ± 0.49

Literature values of metabolites are derived from:^9,13,27,33,34,38^.

### Evaluation of the consistency of HPLC and NMR data

ICC is widely used in inter-rater reliability analyses to test whether different raters have the consistent evaluation for the same subject. An ICC score in the range of 0.50 to 0.71 represent moderate agreement and from 0.71 to 0.90 represent strong agreement^36^. In the ICC results, HPLC and NMR showed strong agreements for propionate, butyrate, isobutyrate and isovalerate. There was a moderate degree of agreement for acetate and valerate (Table 2).

**Table 2:** HPLC and NMR reliability evaluation results of each volatile fatty acids.

ICC (2, A, K)	Auto-scaling
Acetate	0.542*
Propionate	0.833*
Butyrate	0.703*
Isobutyrate	0.741*
Isovalerate	0.714*
Valerate	0.627*

‘2’ represent Two-way random/mixed, ‘A’ represent Absolute agreement, ‘K’ represent Multiple raters/measurements.

‘*’ represents significantly agreement in the ICC two-tail test.

### Comparison of HPLC and NMR results based on the molar proportions of VFA

**Figure 2:**
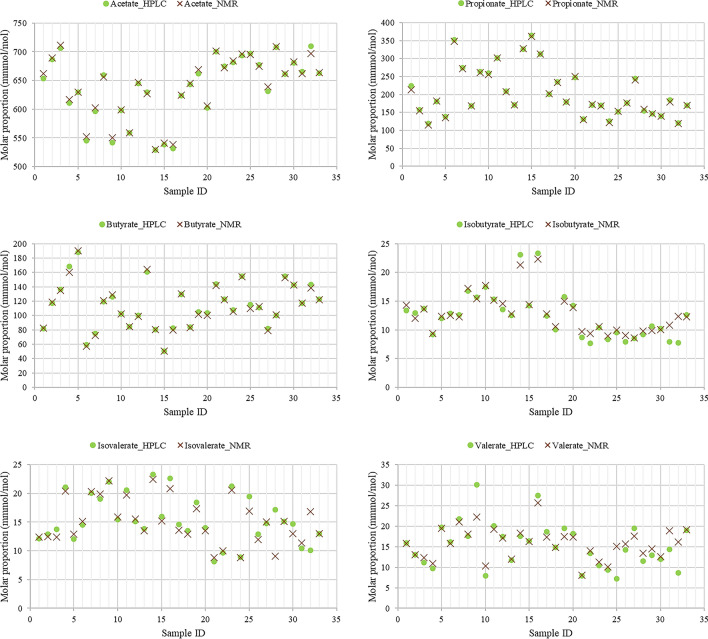
Comparison of HPLC and NMR measurements of individual VFA (mmol/mol) in the same rumen samples.

To compare the data intuitively between HPLC and NMR, the concentrations of VFA were transformed into molar proportions. The molar proportion derived from HPLC and NMR was based on the computation of the relative concentration of each VFA as shown in Fig. 2. Figure 2 represents the individual measurements of VFA (in molar proportions) determined in rumen samples from 33 animals using both HPLC and NMR. Generally, the individual measurements over all samples indicate that the HPLC and NMR data between samples tend to be consistent. Table 3 shows the correlations between VFA (in molar proportion) using HPLC and NMR. The two sets of data (using HPLC and NMR methods) were highly correlated among VFA.

**Table 3:** Correlations between VFA (mmol/mol) measurements determined by HPLC and NMR in units of molar proportion.

Metabolite	Pearson correlation
Acetate	0.9973***
Butyrate	0.9976***
Propionate	0.9993***
Isobutyrate	0.9636***
Isovalerate	0.8743***
Valerate	0.8846***

***P < 0.01 for significance test.

### Comparison of HPLC and NMR experimental procedures

**Figure 3:**
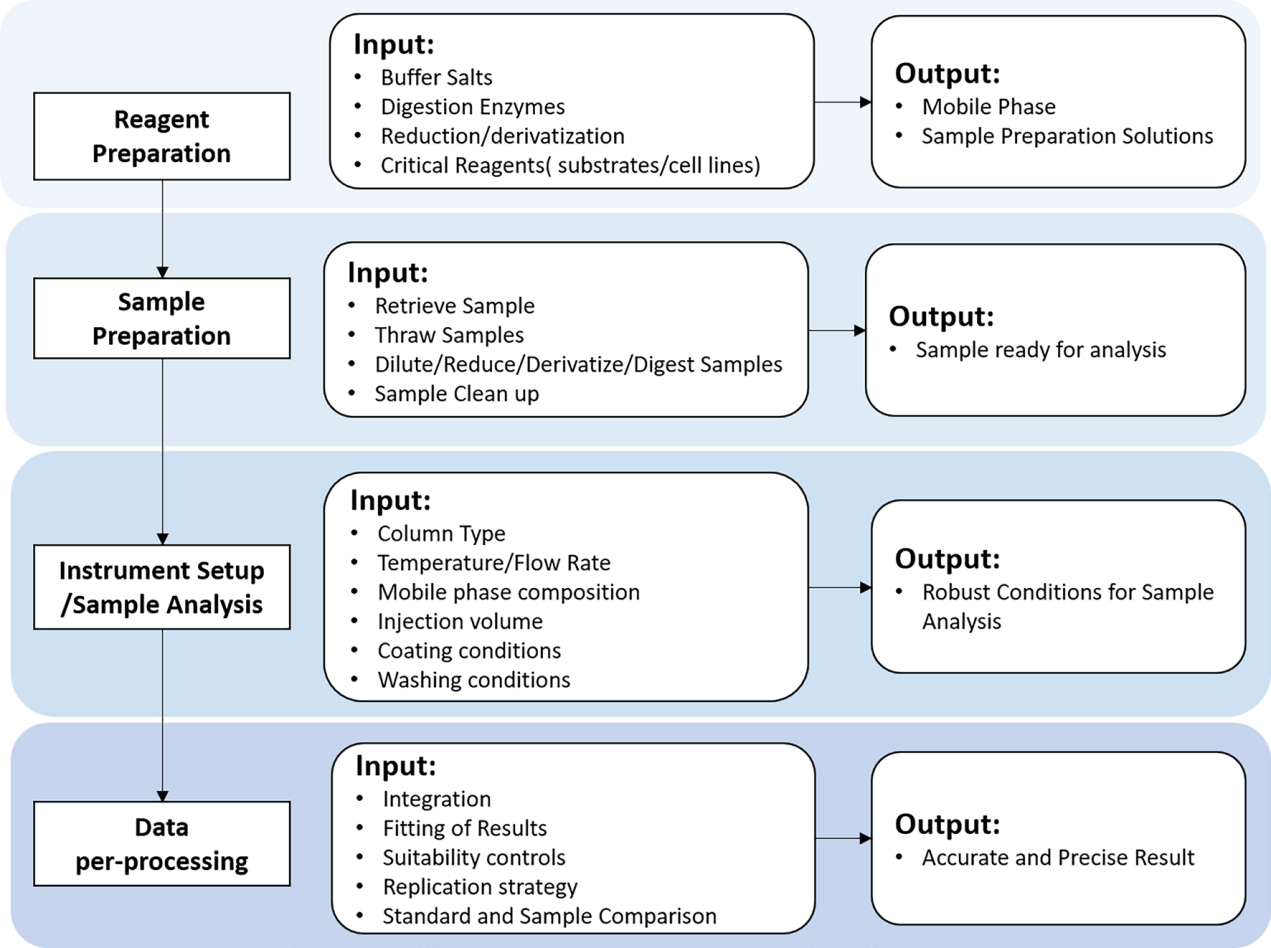
Summary of HPLC experiment workflow.

The differences between the HPLC and NMR experimental procedures for obtaining quantitative concentrations are discussed in this session. The experimental procedures of HPLC are relatively simple mainly including sample preparation, selection of chromatographic conditions (selection of chromatographic column, determination of mobile phase, flow rate, detector, and column temperature, etc.), standard curve preparation, sample determination, sample concentration calculation, and recovery experiment as illustrated in Fig. 3. This differential washout or elution of compounds is the basis for the HPLC separation^10^. Although HPLC is simple to set up and operate, the operator must have a thorough understanding of the system, its columns, and the chemistry of the compounds being separated to achieve optimal separation.

**Figure 4:**
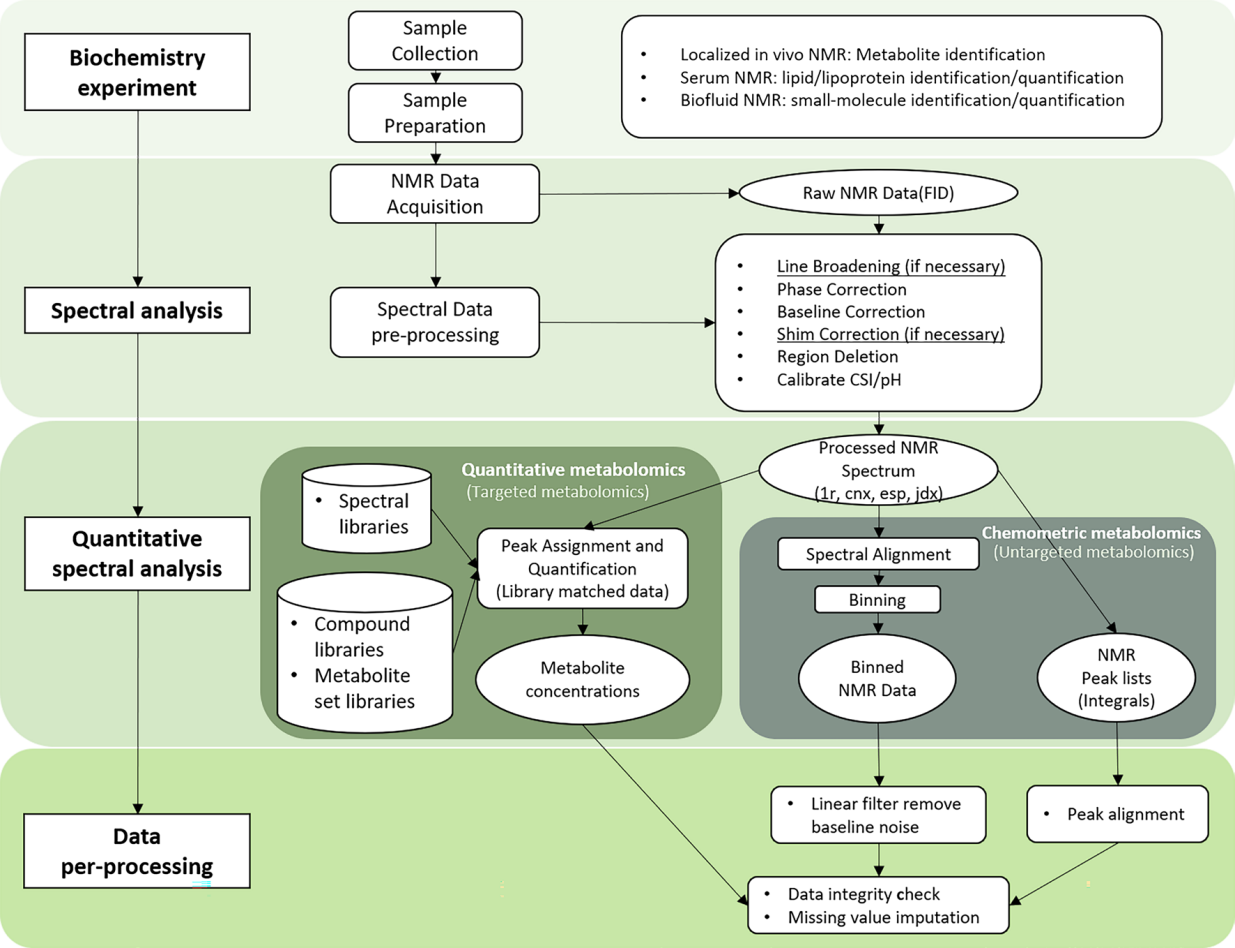
Summary of NMR experiment workflow.

Most metabolites have unique NMR chemical fingerprints consisting of numerous signals^37^. By comparing the integral of the reference peak, which reflects the know concentration of a standard, to the integrals of the sample peaks, it is possible to quantify the metabolites^37^. Careful preparation of spectra for quantitative analysis consists of several steps, including phasing of the spectra, baseline correction and potentially alignment of the signals (Fig. 4). Metabolites are identified and quantified by comparing the bio-NMR sample's spectrum to a set of legitimate standards or a spectral reference library derived from authentic standards. This can involve the acquisition of additional (2D) NMR experiments to identify individual metabolites or comparison with metabolites contained in the Bovine Metabolome Database (BMDB)^33,38^, where currently the details of more than 200 metabolites found in bovine ruminal fluids can be found. An alternative approach to the quantitative analysis of NMR data is an untargeted approach using chemometric analysis (Fig. 4). Chemometric profiling is fundamentally different from quantitative metabolomics (or targeted metabolic profiling) (Fig. 4). Only the spectral patterns and intensities of the chemicals are recorded, statistically compared, and utilised to determine the key spectral characteristics that separate sample classes.

### Summary of NMR spectrum pre-processing and quantitative practice

The use of sophisticated curve-fitting software and specifically created databases of NMR spectra of pure metabolites obtained at appropriate pH levels and spectrometer frequencies is required for spectrum analysis (100–900 MHz) (Fig. 4). In this study, we used Chenomx software to perform spectral pre-processing and subsequent quantitative analysis (targeted metabolomics). The spectrum pre-processing steps and order in this study follow Chenomx's tutorial as shown in Fig. 4. When using Chenomx components to quantify compounds, we found that in the pre-processing steps of spectroscopy, as stated in the official tutorial^39^, phase correction and baseline correction are essential steps, and largely determine the accuracy of quantitative concentration. However, it is worth noting that line broadening and shim correction can make the curve smooth, but if there is no strong evidence that the above steps should be taken, they should be used with caution. Because it was found in the study that the above two steps are likely to significantly reduce the quantified concentration of the overall metabolites. Another point worth emphasizing is as stated in the official tutorial^39^, when determining the true concentrations of compounds with multiple ^1^H signals, some are more reliable in determining the true concentration of the compound than others. Less overlap, undistorted, and more intense signals, representing more protons that are split by fewer *J* couplings, are all signs of a more reliable signal. This study identified signals that are more reliable in the quantification of VFA compounds with multiple ^1^H signals. These are referred to as “handle signals” and presented in Table 4. Quantitative analysis of VFA signals in ^1^H NMR spectra was performed by Chenomx. The ^1^H signals of most compounds such as isobutyrate, isovalerate and valerate, overlap, which makes their quantification difficult. In reference to Table 4, spectra of individual compounds (Fig. S1) are colour coded and triangles indicate their individual ^1^H signals.

**Table 4:** Summary of ^1^H chemical shift of investigated VFAs.

Metabolites	Individual chemical shift (ppm)
Butyrate	2.1 1.5* 0.9
Propionate	2.2* 1.0
Isobutyrate	2.4* 1.1
Isovalerate	2.0 1.9* 0.9
Valerate	2.2 1.5* 1.3 0.9

The numbers in bold and * indicate the chemical shifts of the “handle signal”.

### Comparison of HPLC and NMR quantitative data analysis

The purpose of data pre-treatment is to reduce systematic variation and to separate biological variation from variation introduced during the quantification of metabolites to improve the performance of the downstream statistical analysis. Experimental heterogeneity, such as sample inhomogeneity, variations in sample preparation and ion suppression, account for the unexplained variance in data^40^. Data normalisation is used to eliminate systematic bias within a data set and increase overall data accuracy to make valid biological comparisons^41^. In this section, we discuss several methods recently reported in the literature and test their suitability for VFA analysis.

### Summary of HPLC and NMR data pre-treatment and analysis method

**Figure 5:**
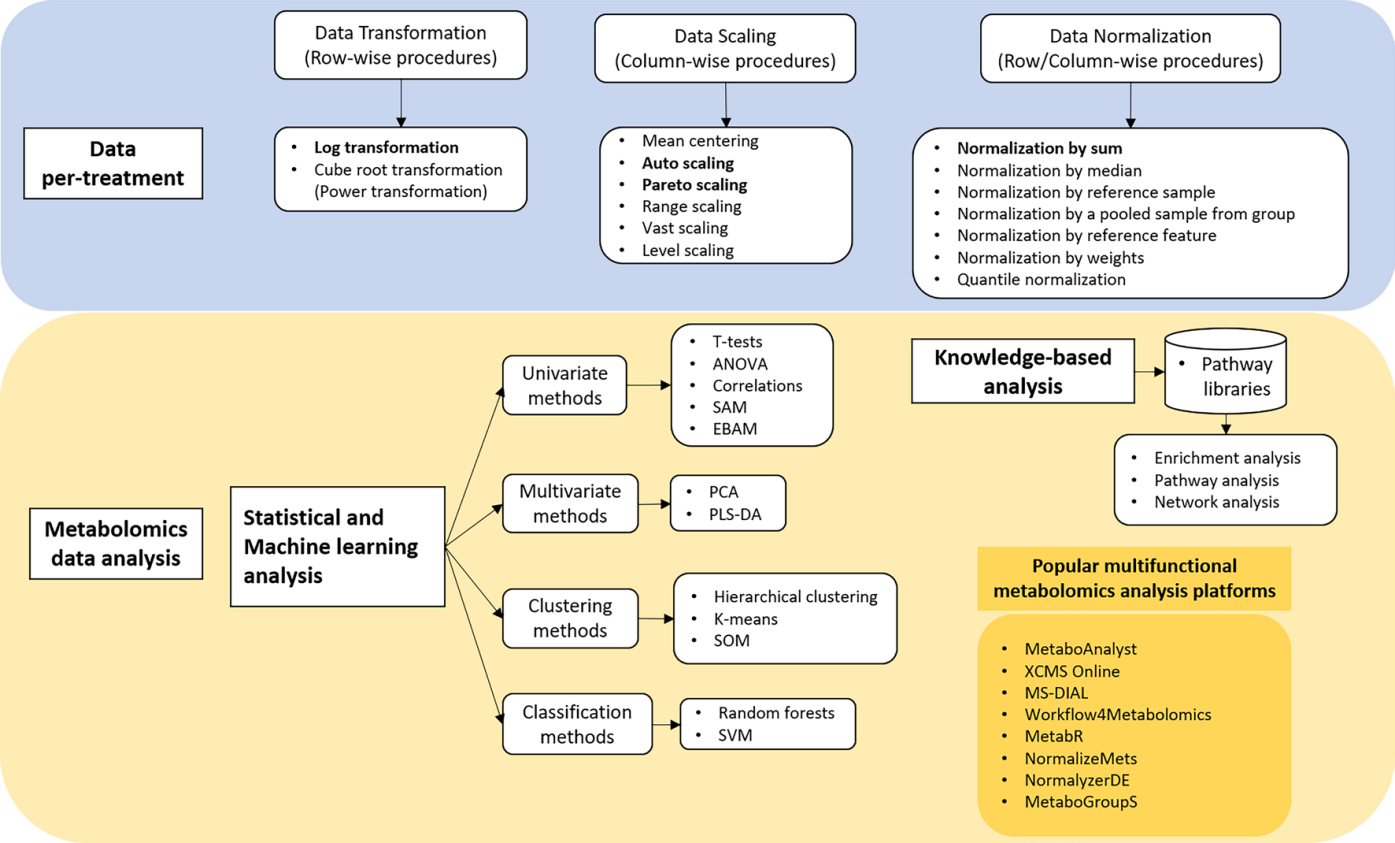
Summary of data analysis using metabolite data.

Data pre-treatment is a crucial ‘key’ step in the metabolomics analysis pipeline, among all the other stages listed above^42^. Figure 5 illustrated several data pre-treatment, downstream analysis and popular functional platforms recommended in the literature^21^. There are three types of normalization: sample/variable normalization, data transformation and data scaling.

1. The normalisation phase is done to each sample's data and consists of methods for making data from all samples directly comparable. One of its most popular applications is to eliminate or reduce the impacts of changing sample dilution^41^. Dilution is described as a process in which the concentrations of all metabolites, and therefore all peak intensities of the associated spectrum, are influenced by the same factor (coefficient), also known as unspecific metabolite alterations. There were three methods of normalising: row-wise, column-wise, and combine normalisation. Normalization by row tries to make each sample (row) comparable to the others (i.e., samples with different dilution effects) (Figs. S3 and S4). Examples include normalization by sum, normalization by the median, and sample-specific normalization.
2. When the data distribution is skewed or asymmetric, it will bring limitations and challenges to the application of statistical analysis. In this scenario, a suitable transformation might be required to transform variable distribution close to the normal or Gaussian distribution^43^. Transformation is also applied to correct for heteroscedasticity. There are two widely used data transformation methods: log transformation and cube-root transformation. Among them, log transformation is the more often used method^44^.
3. Many metabolomics data characteristics (such as chemical concentration or ion abundance) have a large dynamic range. The ranges of variables can be vastly diverse, causing modelling and interpretation issues. The goal of data scaling is to reduce the fold differences between metabolites of various concentration levels so that they may be compared^45^. When the variables are of significantly different orders of magnitude, this approach is beneficial (some metabolites are at micromolar levels while other metabolites are at millimolar levels). Auto-scaling, Pareto scaling, and range scaling are all techniques of scaling (column-wise operation). The standard deviation is used in auto-scaling, whereas the square root of the standard deviation is used in Pareto scaling. For correlation analysis, autoscaling is used more commonly, and the data processed using Pareto scaling is more similar to the original data structure^40^. Column-wise technique, in contrast to row-wise process, aims to make each variable (column) equivalent to the others (Figs. S3 and S4)^46,47^

### Biological interpretability analysis of HPLC and NMR

In this section, VFA data derived from HPLC and NMR after applying different data pre-treatment were compared and analysed. The two sets of data are expected to show the same biological interpretability in the correlation, PCA analysis and PLS-DA analysis results.

**Figure 6:**
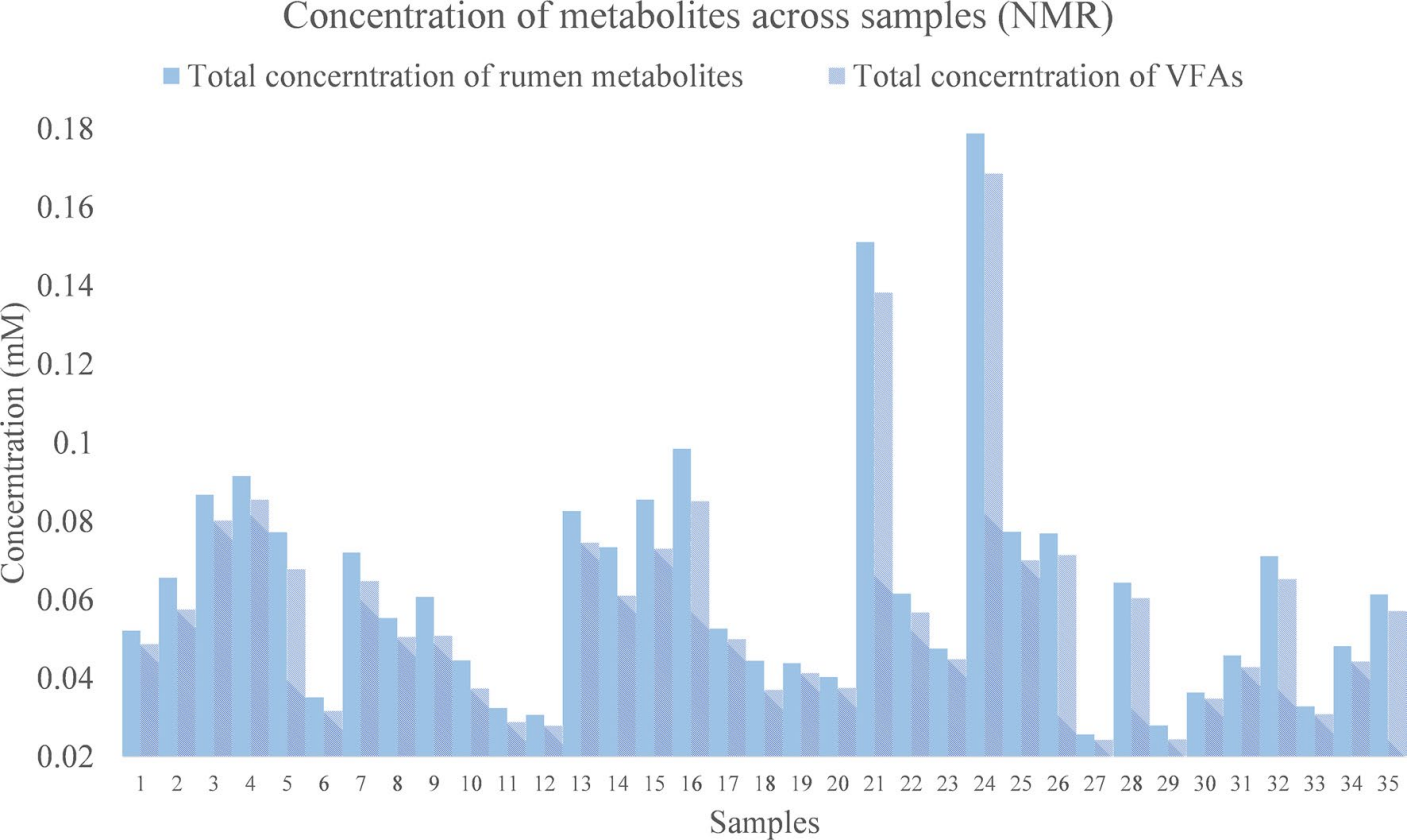
Comparison of total metabolite concentration and VFA concentration in NMR analysis results.

The overall sample quantity or metabolite concentration might change considerably among samples when using NMR-based values (Fig. 6). It's critical to minimise or eliminate the impact of total sample variance on individual metabolite measurement. However, we must consider the degree to which they are affected in various analyses, as well as the extent to which data pre-treatment will mitigate these effects. The experimentally obtained HPLC data are in the type of molar proportion, which means the total VFA content are all approximately equal to 1. In this case, there is no comparison of the total rumen volatile fatty acids.

#### Pearson correlation analysis

**Figure 7:**
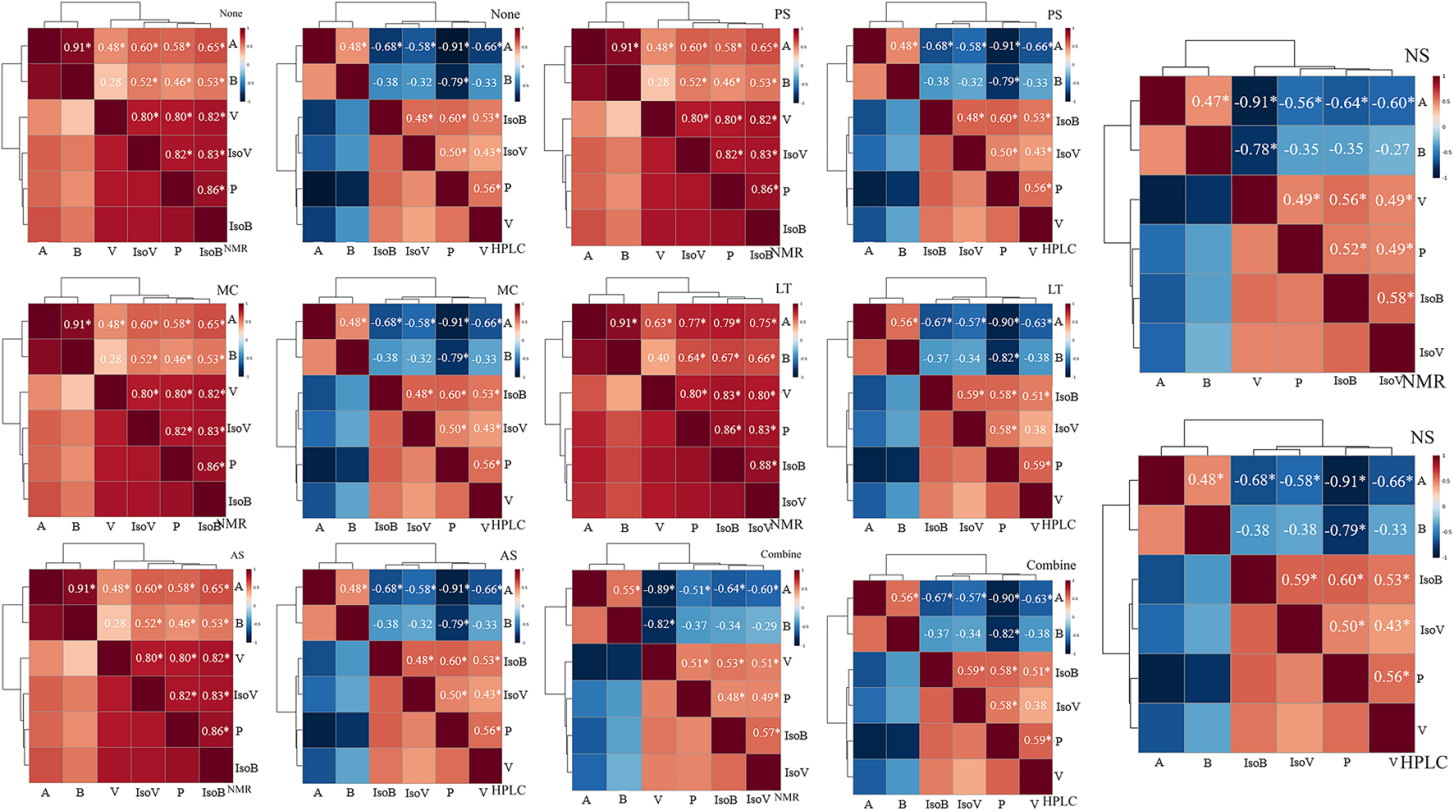
Correlation heatmaps comparison of HPLC and NMR metabolite data. Pearson correlation heatmaps of the NMR and HPLC data of VFAs under six different pre-treatments, including MC, AS, PS, LT, NS and Combine. None represents data without pre-treatment. The blue colour indicates that the correlation coefficient is close to − 1, and the red colour indicates that the correlation coefficient is close to 1. The lines on the heat map indicate hierarchical clustering results. *Indicates a significant correlation, *p* < 0.05. ‘A’ represents Acetate. ‘B’ represents Butyrate. ‘P’ represents Propionate. ‘V’ represents Valerate. ‘IsoB’ represents Isobutyrate. ‘IsoV’ represents Isovalerate.

Pearson correlation is one of the basic measures for the downstream analysis (i.e., network analysis), so this study investigated the impact of different data pre-treatment methods on the results of correlation analysis. For correlation analysis, HPLC data showed results consistent with biological knowledge, that is, acetate and butyrate were positively correlated, and propionate was negatively correlated, and the hierarchical clustering results also showed that acetate and butyrate were classified into one category based on correlation (Fig. 7). The data processing of scaling (MC, AS, PS) and NS did not have any effect on the results of the correlation matrix (Fig. 7).

For correlation analysis, the batch effects of NMR technical artefacts together with effects from standardization may exhibit inflated variation between samples resulted in large positive correlations^46^. As illustrated in Fig. 7, NMR data under pre-treatments including None, MC, AS, PS, and LT showed unusual positive correlations. This is also inconsistent with biological knowledge, for example, propionate and acetate are usually negatively correlated. In a previous study based on NMR data, the large positive correlations in the non-normalized data have also been observed^42^. In general, both negative and positive correlations should be present, rather than such large positive correlations of NMR data which obviously cannot reflect reality. The problem might be caused by batch effects or the calibration standard. The requirement for metabolomics data normalisation is critical in this case.

In the research results, NS and Combine pre-treatment the NMR data showed positive and negative correlations consistent with HPLC, and the hierarchical clustering results also divided acetate and butyrate into one category, and other VFA into another category, which also agrees with the biological knowledge (Fig. 7). To eliminate variance caused by causes other than homeostatic changes, normalisation is required. Both the HPLC and NMR data showed improved concordance after normalisation, and there were no significant discrepancies in calculated correlations. The inclusion of a random sample effect in the normalisation is most likely to eliminate the impact of the calibration standard. It was found that after scaling (MC, AS, PS) was applied, the correlation matrix of HPLC and NMR did not change. Log conversion adjusted correlation coefficients but did not eliminate the large positive correlations in the NMR data. Positive correlations were stronger in the standardized data, which is concerning.

#### PCA analysis

**Figure 8:**
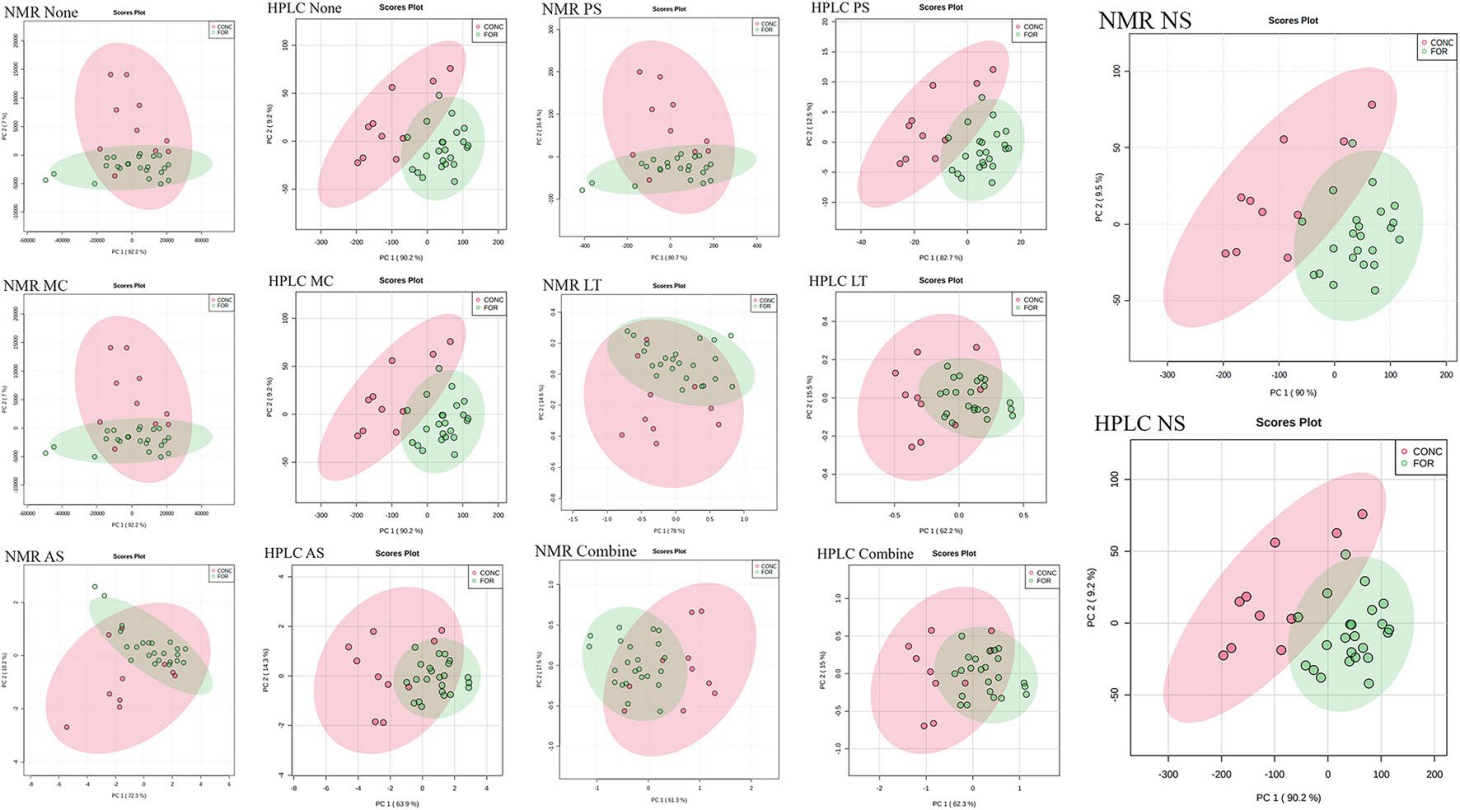
PCA analysis comparison of HPLC and NMR data. PCA analysis of the NMR and HPLC data of VFAs under six different pre-treatments, including MC, AS, PS, LT, NS and Combine. None represents data without pre-treatment. X-axis represents the first principal component. Y-axis represents the second principal component. Dots represent the sample. CONC represents the concentrate diet, FOR represents the forage diet. The shading of the ellipse indicates 95% confidence interval. The direction of the long and short axis of the error ellipse should be direction of the eigenvector of covariance, and magnitude is equal to the eigenvalue.

For PCA analysis, the first two principal components were selected to present the results. Under different pre-treatments, 74.1%-99.5% of metabolite-based phenotypic variation can be explained. For NMR data, only the pre-treatment of NS maximized the 95% confidence interval difference between the two groups of CONC and FOR diet samples (Fig. 8). And the explanation of phenotypic variation under NS treatment is also the highest among all pre-treatment results, reaching 99.5% (Fig. 8). For the HPLC data, the original data, MC, and NS pre-treatment explained 99.4% of the phenotypic variation and achieved similar characteristic values with NMR (NS), including direction and size (Fig. 8). In the phenotypic-related PCA exploratory analysis, both HPLC and NMR data were processed by NS to extract similar high-interpretation feature values. Because PCA seeks to explain as much variation as possible in as few components as feasible, whereas correlation focuses on the investigation of (dis)similarities. Using data pre-treatment to alter data attributes may thus improve the outcomes of correlation approach while blurring the feature variance of PCA analysis^22^.

#### PLS-DA analysis

**Figure 9:**
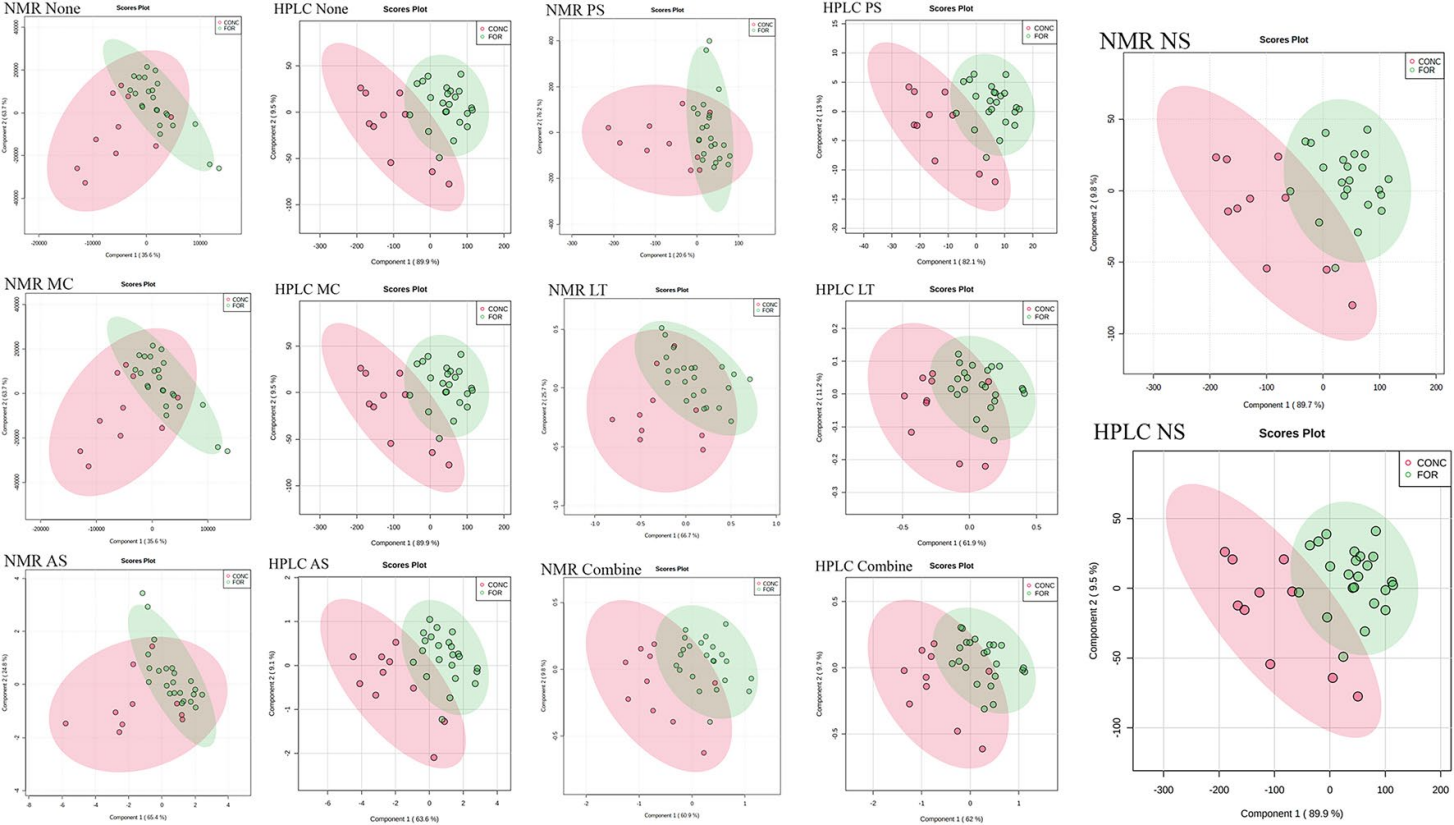
PLS-DA analysis comparison of HPLC and NMR data. PLS-DA analysis of the NMR and HPLC data of VFAs under six different pre-treatments, including MC, AS, PS, LT, NS and Combine. None represents data without pre-treatment. X-axis represents the first principal component. Y-axis represents the second principal component. Dots represent the sample. CONC represents the concentrate diet, FOR represents the forage diet. The shading of the ellipse indicates 95% confidence interval. The direction of the long and short axis of the error ellipse should be direction of the eigenvector of covariance, and magnitude is equal to the eigenvalue.

For the PLS-DA analysis of HPLC and NMR data, the first two main components under each pre-treatment can explain 71.1–99.5% of the variation of diet-based samples (Fig. 9). The first two principal components extracted from the NMR data under NS processing in the PLS-DA analysis explained 99.5% of the phenotypic variation and achieved the highest R^2^ and Q^2^ in cross-validation (Fig. S6). Comparing all the two components model, HPLC achieved the R^2^, Q^2^ and accuracy greater than 0.6 in None, MC, PS, NS and Combine which indicated the prediction and fitting effects of these models are good. NMR data have the best combination of R^2^, Q^2^ and accuracy under NS pre-treatment. And in the PLS-DA results of NMR data, only NS has the 95% confidence interval of different diet samples based on extracted features that are well separated (Fig. 9). For HPLC data, the data under LT and Combine reduced the extracted feature values of PLS-DA and blur the differences between phenotypes. Finally, based on the NMR and HPLC data of NS, the results of PCA and PLS-DA analysis have obtained the two most explanatory principal components and can maximize the diet-based phenotypic differences, which is also consistent with biological knowledge.

As a summary in Table 5 showed, pre-treatment that has a positive impact on the analysis results should be given priority. Pre-treatment that has no impact is acceptable, but pre-treatment that has a negative impact should be carefully used. Scaling makes it possible to compare the variability between samples based on the same metabolite. This type of data processing has achieved good results in some case studies, especially for reflecting the accurate rank of metabolites^22^. However, in our study, it is also very likely that there are only a limited number of metabolites in the VFA data, which does not reflect the magnitude of differences between metabolites. Therefore, scaling does not play a positive role in the specific scenario with a lack of fold difference^42^. In similarity-based analysis, such as clustering or correlation, the Combine normalization approach performing predefined row- and column-wise procedures was found to work best with each other^31^. Our research showed that the Combine method does not perform well in PCA and PLS-DA analysis which indicated complicated data pre-treatments are likely to over-eliminate some important biological differences. NS can highlight the variation of the same metabolite between different samples. Normalization to total intensity is the most common method that showed a superior role by reducing the sample variability due to differences in sample concentrations.

**Table 5:** Summary of the influence of pre-treatments on correlation, PCA and PLS-DA analysis.

Dataset	Pre-treatment	Pearson correlation		PCA	PLS-DA
		Between metabolites	Between samples		
Absolute concentration (NMR)	NS (row-wise)	P	NA	P	P
	LT (row-wise)	P	NA	N	N
	MC (column-wise)	NA	P	NA	NA
	AS (column-wise)	NA	P	N	N
	PS (column-wise)	NA	P	N	N
	Combine	P	P	N	N
Proportional data (HPLC)	NS (row-wise)	NA	NA	NA	NA
	LT (row-wise)	P	NA	N	N
	MC (column-wise)	NA	P	NA	NA
	AS (column-wise)	NA	P	N	N
	PS (column-wise)	NA	P	NA	N
	Combine	P	P	N	N

‘P’ indicates that the pre-treatment has a positive effect on the analysis results of the set of data. 'N' The pre-treatment worsened the analysis results of the set of data and had a negative impact. The pre-treatment of 'NA' did not affect the analysis results of the set of data.

In the downstream analysis results of NMR and HPLC, in the hierarchical clustering based on Pearson correlation, acetate and butyrate are found in one category, whilst isobutyrate, isovalerate, propionate and valerate are found in another. This is in line with the established knowledge about propionic acid production increasing under concentrate feeding and acetate production increasing under forage feeding. The consistency of the two sets of NMR and HPLC data in PCA and PLS-DA also shows that diet can explain more than 90% of the variation in the composition of VFAs. The Table S1 provides the standard deviation of the concentration of each VFA under the FOR and CONC diets, which shows that majority of data from FOR diet are less divergent. The regulation of rumen digestion and metabolism through diets varies greatly due to individual differences in cattle. The production and ratio of VFA are not only related to the feed itself, but also affected by other factors, including minerals, ionophores, animal age and feeding time, organic matter, outflow rate, enzyme preparations, health conditions, etc. The CONC diet may cause more uneven changes in volatile fatty acids in the rumen due to its faster fermentation.

## Conclusion

In this study, HPLC analysis and NMR targeted metabolomics produced consistent results in the quantification of VFA. For the molar proportion comparison and results of the ICC index, there are still deviations for low-concentration metabolites, but the trends of the data across the samples are always consistent (Table 3), which means that useful information can be provided for downstream analysis. Both techniques can attain the same level of accuracy. Most of the HPLC equipment is automated. The analysis steps are relatively simple, but most of them are used for the content analysis of several target metabolites. If researcher wants to perform fingerprint analysis of metabolomics, it needs to be in conjunction with other technologies, such as mass spectrometry.

Compared with the HPLC method, NMR-based quantitative metabolomics is capable to identify and quantify all detectable metabolites from any given sample spectrum which is the sum of individual spectra from each of the mixed metabolites. Quantification of metabolites needs the support of sophisticated curve-fitting software and databases of NMR spectra of pure metabolites collected at specific spectrometer frequencies. For the Chenomx component processing spectrum, this research gives the following suggestions:

1. Line broadening and shim correction as unnecessary pre-processing steps need to be used with caution unless there are sufficient evidence.
2. Different ^1^H signals of one compound have different importance in quantification. The principles of less overlap, more protons, less couplings and avoiding distortion can be referred to VFA signals used for quantification in this study are identified as “handle signals” and are presented in Table 4.

Results were subject to a combination of technical and biological variability. Only when data pre-treatment is motivated by deep knowledge and significant attention to uninduced biological and analytical variance can it address features that hinder biological interpretation^47,48^. Every biological researcher must be vigilant about errors introduced by data pre-treatment. There is no consensus about which approaches work best for different types of metabolomics data^31,49–51^.

The following are some practical recommendations based on our analysis:

1. NMR concentration data is likely to have a large area of positive correlations (Fig. 6)^40^. For Pearson correlation, NS as row normalization made the correlation between metabolites consistent with biological knowledge. Scaling (MC, AS, PS) as column transformation produces correlations between samples with biological meaning (Fig. S5). On the contrary, NS does not have any effect on the correlation between rows (sample), and scaling does not have any effect on the correlation between columns (metabolite/variable).
2. LT conversion slightly adjusted correlation coefficients in the range of ± 0.08 and the direction of correlation (positive or negative correlation) were not changed.
3. For correlation analysis, the Combine pre-treatment worked best for all metabolites and samples, including for both relative and absolute concentrations. In the case that the relative data itself can present reliable correlations, there is no need to worry that Combine pre-treatment will distort the biological significance of the results. For some pipeline-based analysis platforms, one-time data pre-treatment can be directly applied to a variety of different downstream analyses. Under the premise of no negative impact, there is no need to go back to the original data pre-treatment step repeatedly, which can improve the analysis effectiveness.
4. Different from correlation analysis, for the analysis of extracted feature variables such as PCA and PLS-DA, NS is recommended, and other pre-treatments need to be used with caution. To obtain the most reliable biological analysis results, it is always necessary to consider complex data pre-treatment that might mask variation present in the original data.

We compared the downstream analysis performance of rumen VFAs obtained by HPLC and NMR under different data pre-treatments. Bovine rumen VFAs are the main component of rumen metabolites, and fundamental indexes to measurements of the metabolic function of the rumen. This result illustrates the general applicability and feasibility of the two methods for analysing rumen metabolites. The highly consistent results in analysis results of these two sets of data illustrate the potential of data pre-treatment to deal with both relative and absolute types of data. Future studies are also expected to repeat the method on more metabolite data and larger data sets to further verify its reliability. In summary, this study provides a comparison of the complete flow of the two experiments and suggestions for countermeasures to possible problems currently encountered.

## Supplementary Information

Supplementary Information.

## Data Availability

Data are available only upon agreement with the agriculture organization and should be requested directly from the authors.
