# Supplementary material for: Comparison of HPLC and NMR for quantification of the main volatile fatty acids in rumen digesta
Source: Sci Rep. 2021 Dec 21;11:24337. doi: 10.1038/s41598-021-03553-9 (PMC8692319; doi:10.1038/s41598-021-03553-9)
Supplement: Supplementary file 1 — Supplementary Information. [file 41598_2021_3553_MOESM1_ESM.pdf]

Comparison of HPLC and NMR for quantification of the main volatile fatty acids in rumen digesta

Mengyuan Wang[1], Haiying Wang[1], Huiru Zheng[1*], Dusan Uhrin[2], Richard J. Dewhurst[3], Rainer Roehe[3]

[1] School of Computing, Ulster University, United Kingdom
[2] School of Chemistry, University of Edinburgh, United Kingdom
[3] Scotland's Rural College, Edinburgh, United Kingdom

*Corresponding author: Huiru Zheng
E-mail addresses: h.zheng@ulster.ac.uk

Supplementary

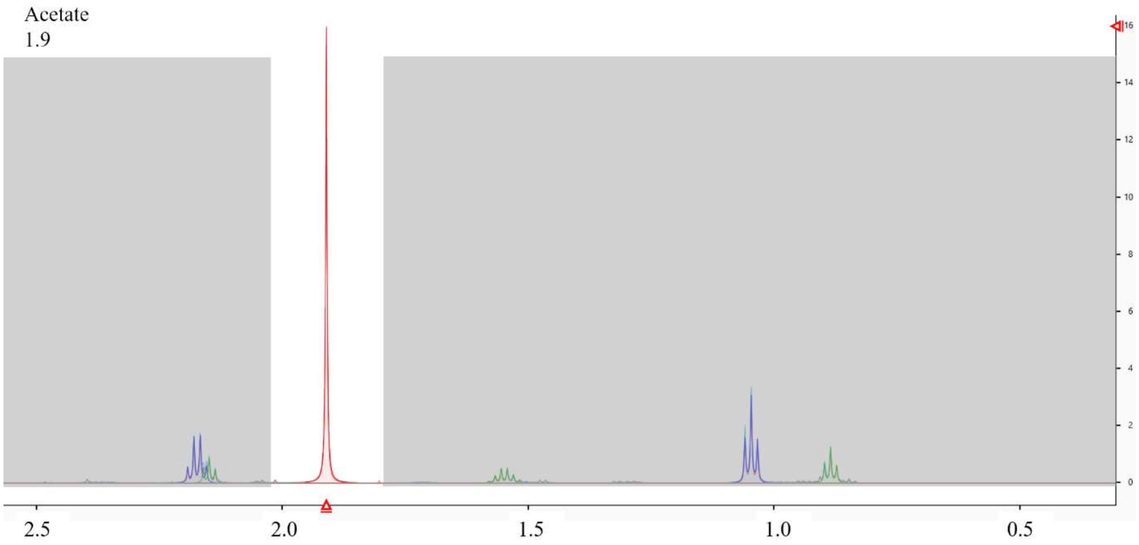

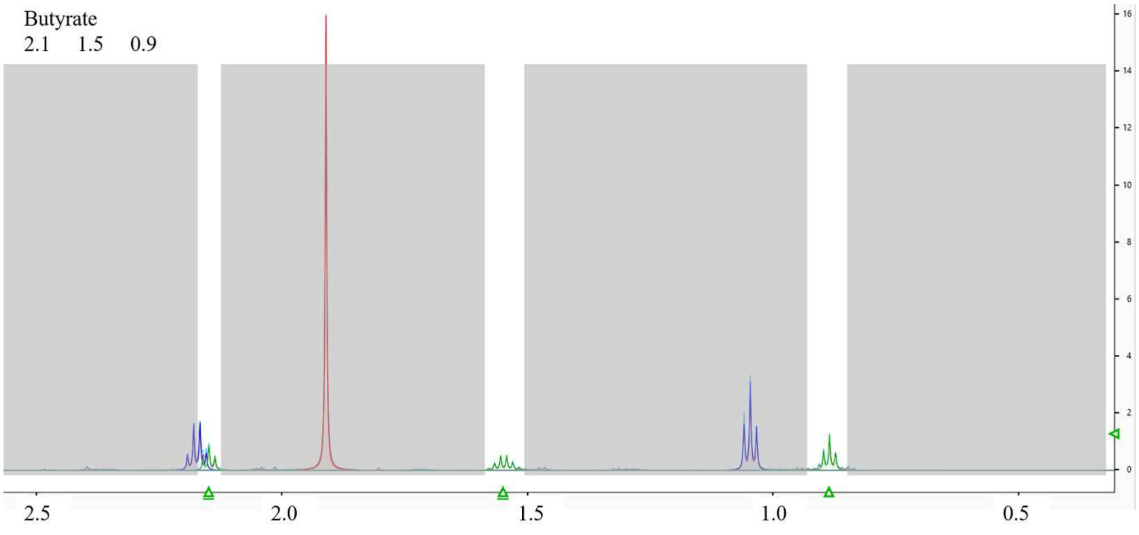

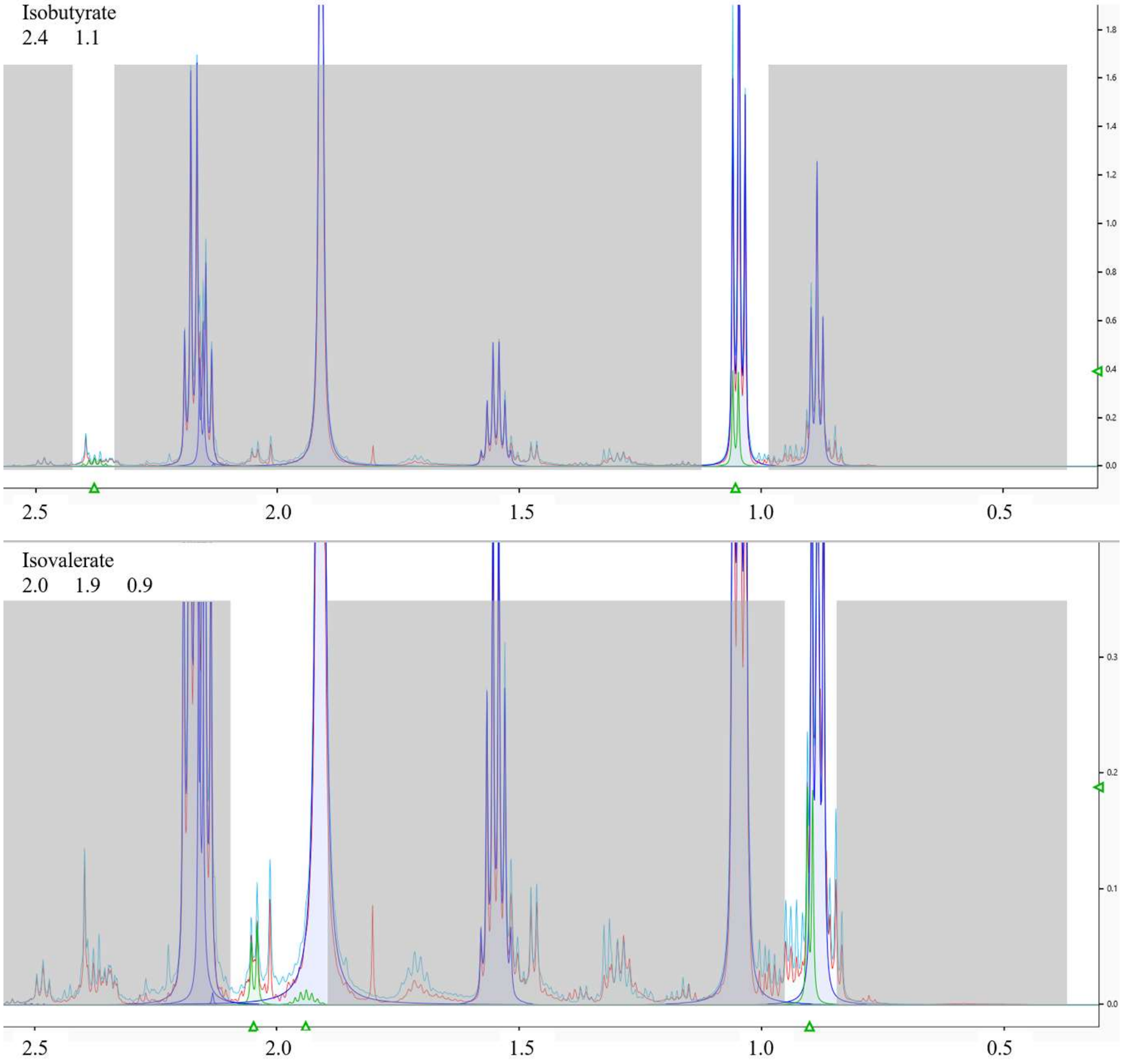

Isobutyrate
2.4 1.1
2.5 2.0 1.5 1.0 0.5
Isovalerate
2.0 1.9 0.9
2.5 2.0 1.5 1.0 0.5

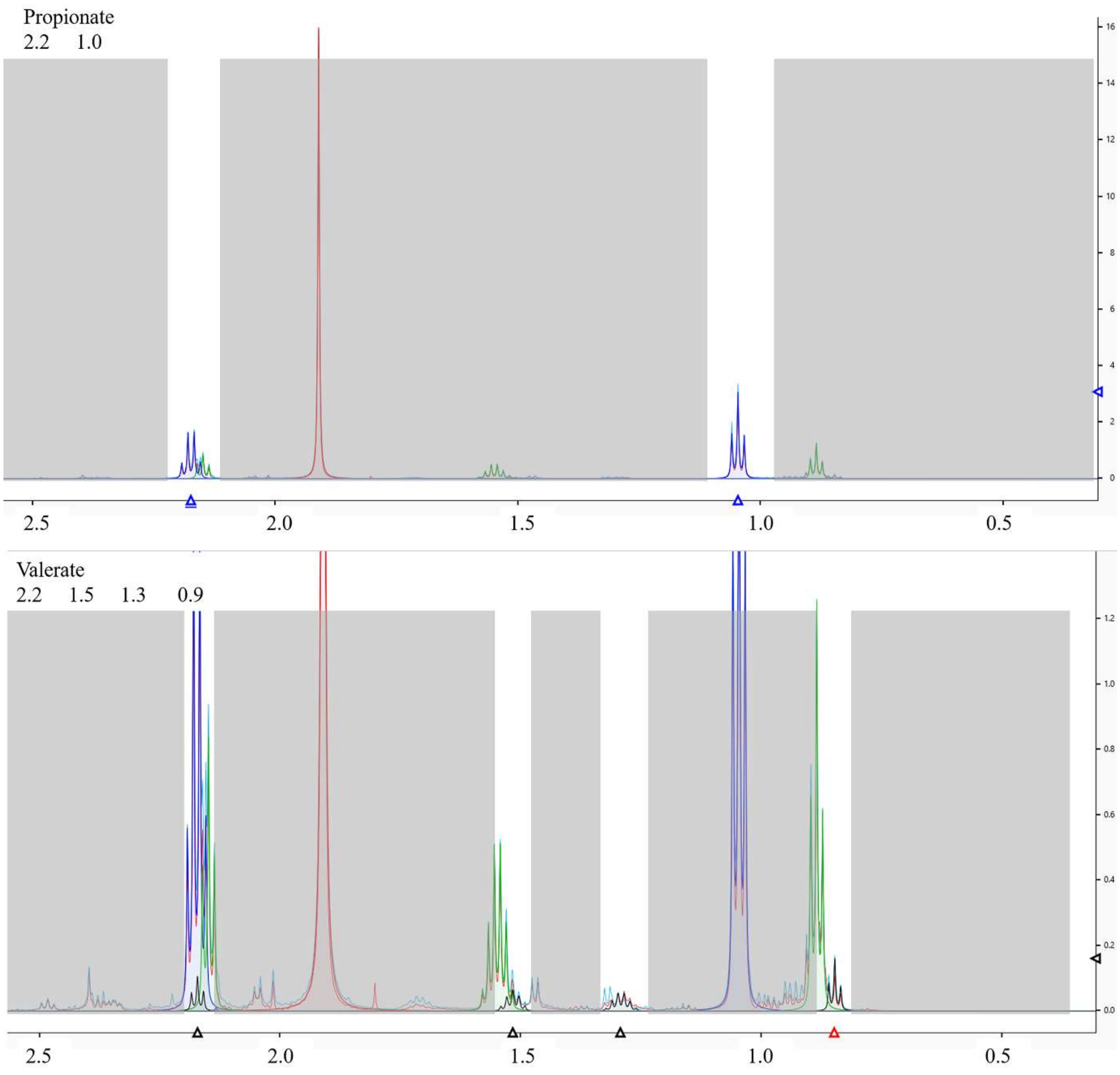

Fig S1. NMR spectrum of VFAs (The abscissa indicates the chemical shift of the compound peak. The peak pointed by the triangle on the abscissa is the target metabolite. All peaks of the same metabolite are given the same colour. The colour of the triangle indicates the "handle signals" that were used for quantification. Specific colour indications: Acetate-Red, Propionate-Blue, Butyrate-Green, Valerate-Black, Isobutyrate-Green, Isovalerate-Green.)

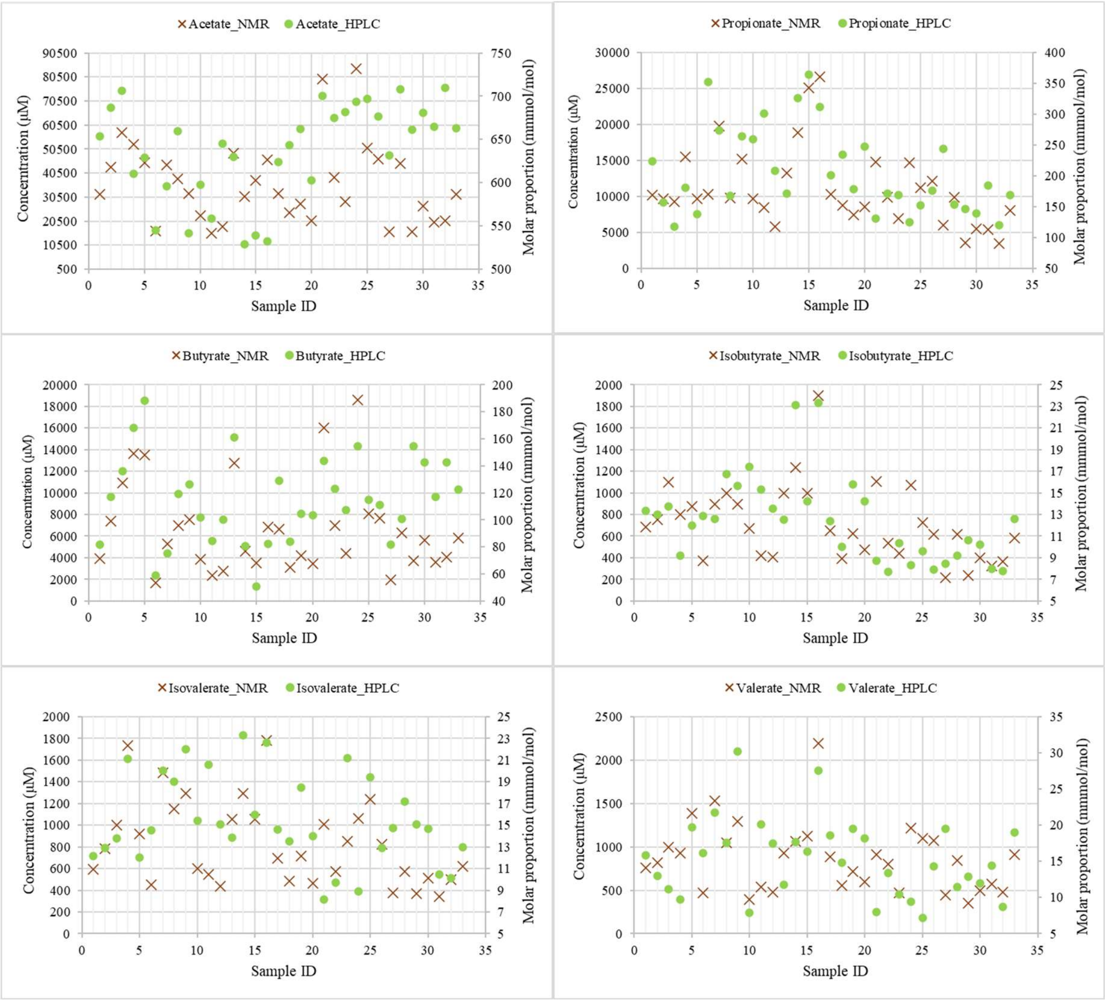

Fig. S2 Comparison of HPLC (mmol/mol) and NMR (Mm) of VFAs in the same rumen samples

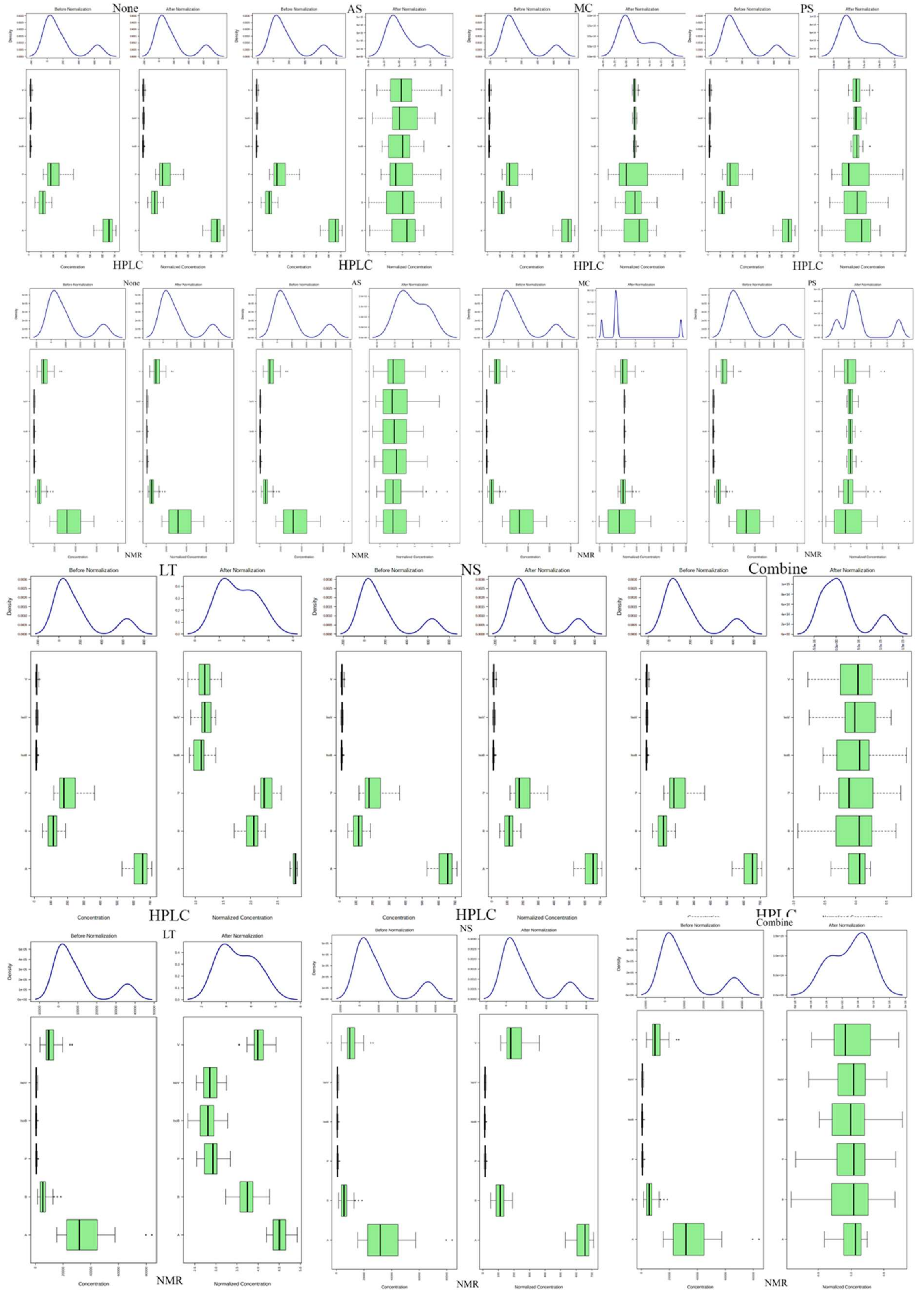

Fig. S3 Data pre-treatment comparison of variable distribution Data pre-treatment results of the NMR and HPLC data of VFAs under six different pre-treatments, including MC, AS, PS, LT, NS and Combine. None represents data without pre-treatment. Line plots show the overall data distribution based on kernel density estimation, before normalization—on the left, and after normalization—on right, while the two horizontal box plots on the bottom show the distributions of individual variables or metabolite concentrations, before and after normalization

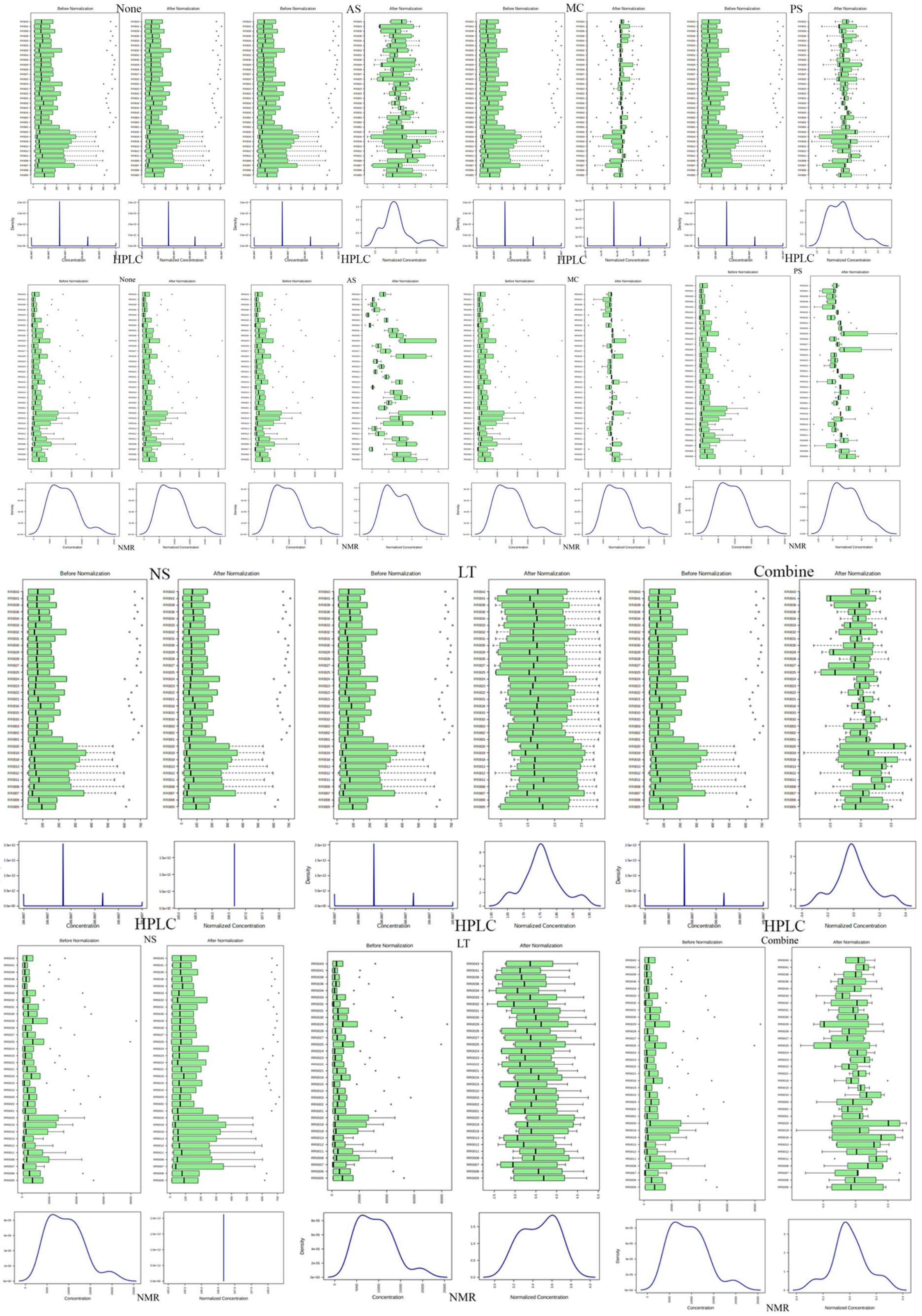

Fig. S4 Data pre-treatment comparison of sample distribution Data pre-treatment results of the NMR and HPLC data of VFAs under six different pre-treatments, including MC, AS, PS, LT, NS and Combine. None represents data without pre-treatment. Line plots show the overall data distribution based on kernel density estimation, before normalization—on the left, and after normalization—on right, while the two horizontal box plots on the bottom show the distributions of individual variables or metabolite concentrations, before and after normalization

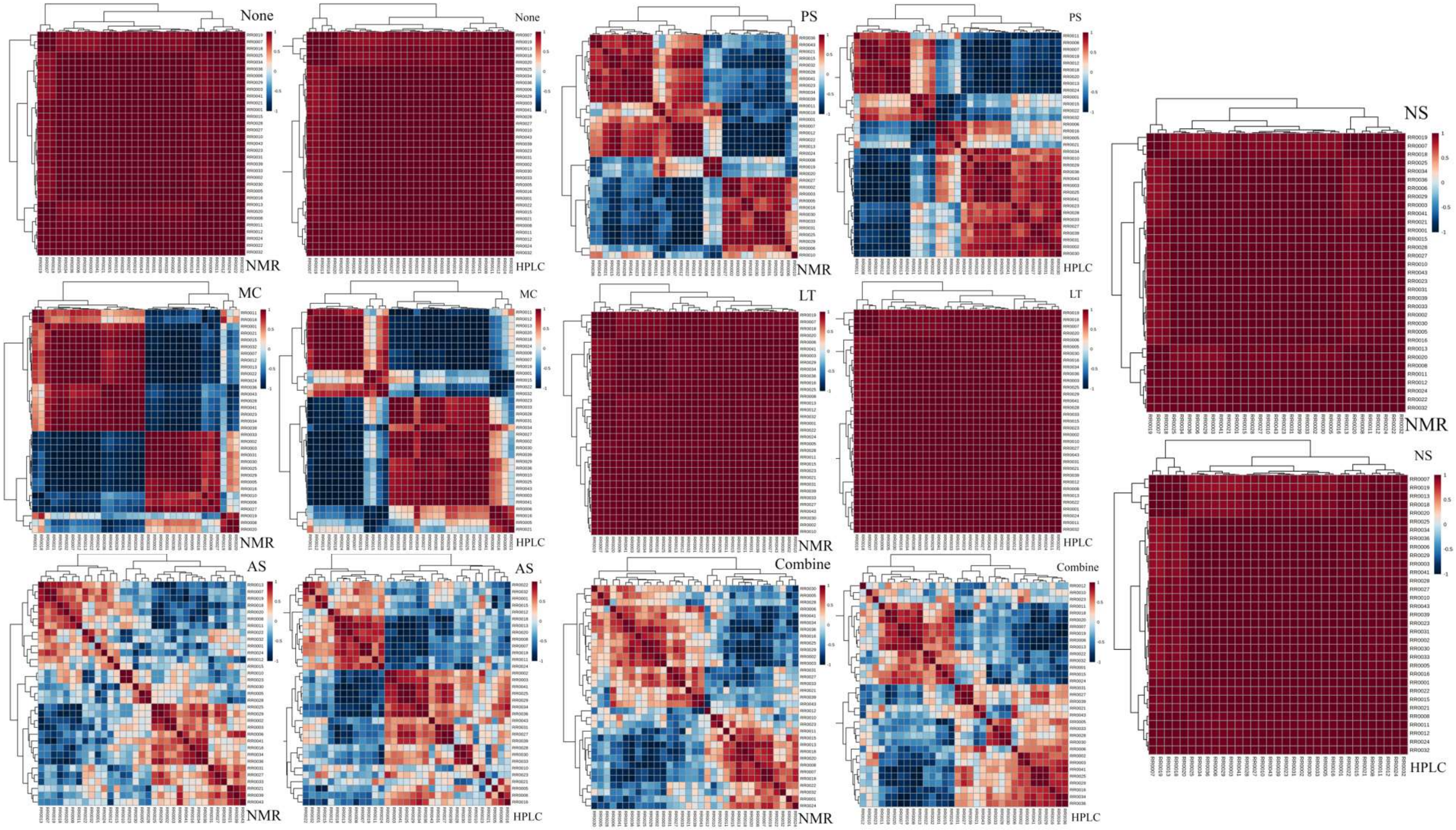

Fig. S5 Correlation heatmaps comparison of HPLC and NMR sample data Pearson correlation heatmaps of the NMR and HPLC data of VFAs under six different pre-treatments, including MC, AS, PS, LT, NS and Combine. None represents data without pre-treatment. The blue colour indicates that the correlation coefficient is close to 1, and the red colour indicates that the correlation coefficient is close to -1. The lines on the heat map indicate hierarchical clustering results. * Indicates a significant correlation, $p<0.05$

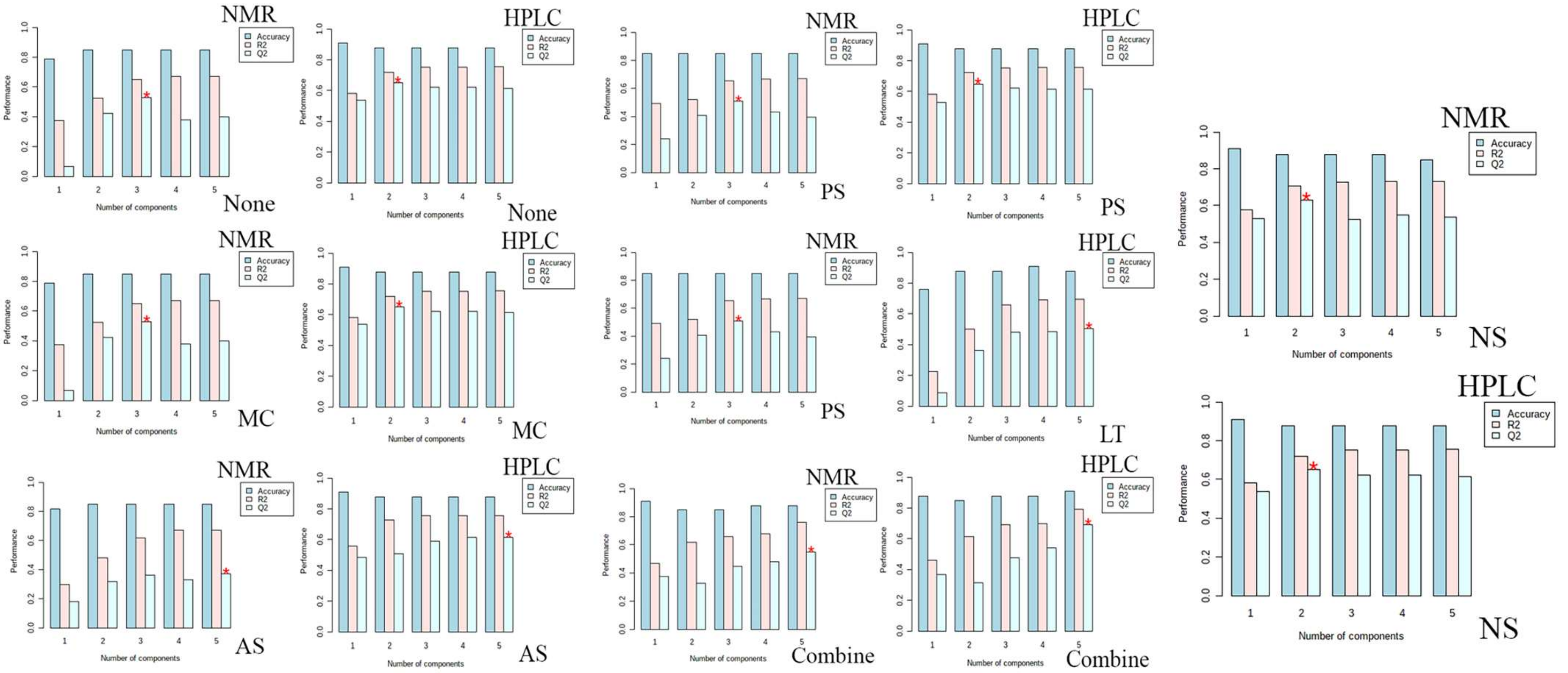

Fig. S6 PLS-DA analysis Cross-validation results PLS-DA analysis Cross-validation results of the NMR and HPLC data of VFAs under six different pre-treatments, including MC, AS, PS, LT, NS and Combine. None represents data without pre-treatment. The sum of squares captured by the model-R^2, the cross-validated Q^2, and the prediction accuracy-Accuracy. The default criterion is Q^2, red star indicates that the component model is the best. PLS-DA performs 10-fold cross-validation on the top five principal components.

Table S1 Standard deviation of VFAs under different dietary treatments

Method	Diet	Acetate	Butyrate	Propionate	Isobutyrate	Isovalerate	Valerate
HPLC	CONC	36.8933	45.6551	71.7547	4.6103	3.9187	6.9207
	FOR	29.2026	23.2207	38.0156	2.6629	3.2628	3.6536
NMR	CONC	13.0181	4.1749	6.2733	0.3964	0.2482	0.3970
	FOR	25.8651	4.0521	3.5211	0.8306	0.1724	0.2855
